# Inhaled Nitric Oxide Reduces Endothelial Activation and Parasite Accumulation in the Brain, and Enhances Survival in Experimental Cerebral Malaria

**DOI:** 10.1371/journal.pone.0027714

**Published:** 2011-11-16

**Authors:** Lena Serghides, Hani Kim, Ziyue Lu, Dylan C. Kain, Chris Miller, Roland C. Francis, W. Conrad Liles, Warren M. Zapol, Kevin C. Kain

**Affiliations:** 1 SA Rotman Laboratories, McLaughlin-Rotman Centre for Global Health, Tropical Disease Unit, Division of Infectious Diseases, Department of Medicine, University Health Network–Toronto General Hospital, and University of Toronto, Toronto, Canada; 2 Department of Respiratory Medicine, Faculty of Medicine, University of British Columbia, Vancouver, Canada; 3 Anesthesia Center for Critical Care Research, Massachusetts General Hospital, Boston, Massachusetts, United States of America; Burnet Institute, Australia

## Abstract

The host immune response contributes to the onset and progression of severe malaria syndromes, such as cerebral malaria. Adjunctive immunomodulatory strategies for severe malaria may improve clinical outcome beyond that achievable with artemisinin-based therapy alone. Here, we report that prophylaxis with inhaled nitric oxide significantly reduced systemic inflammation (lower TNF, IFNγ and MCP-1 in peripheral blood) and endothelial activation (decreased sICAM-1 and vWF, and increased angiopoeitin-1 levels in peripheral blood) in an experimental cerebral malaria model. Mice that received inhaled nitric oxide starting prior to infection had reduced parasitized erythrocyte accumulation in the brain, decreased brain expression of ICAM-1, and preserved vascular integrity compared to control mice.

Inhaled nitric oxide administered in combination with artesunate, starting as late as 5.5 days post-infection, improved survival over treatment with artesunate alone (70% survival in the artesunate only vs. 100% survival in the artesunate plus iNO group, p = 0.03). These data support the clinical investigation of inhaled nitric oxide as a novel adjunctive therapy in patients with severe malaria.

## Introduction

Cerebral malaria (CM) is a severe complication of *Plasmodium falciparum* infection, characterized by coma and convulsions [1]. CM is associated with a high mortality rate, and with long-term cognitive and neurological deficits in survivors [1]–[2]. Although artemisinin-based therapy has reduced the mortality rate associated with severe malaria, the fatality rate associated with CM has remained high (approximately 30% in adults and 18% in children) [3]–[4]. It has long been recognized that the host immune response plays an important role in modulating pathology in malaria, and this has fueled the search for effective immunomodulatory adjunctive therapies [5]. Unfortunately, adjunctive therapies have yet to definitively improve clinical outcome [5]–[6]. A detailed understanding of the mechanisms underlying CM may facilitate the rational design of more effective interventions.

CM is a complex multisystem disorder that is incompletely understood. The accumulation of parasitized erythrocytes (PEs) within the cerebral microvasculature and an excessive inflammatory response to infection are hallmarks of CM [7]–[9]. Fundoscopy and histopathological studies of CM cases have demonstrated sequestration of PEs in the brain, along with perfusion abnormalities (including vascular occlusion), hemorrhages, and local tissue hypoxia and ischemia [1], [10]–[13]. Elevated levels of inflammatory cytokines have been observed in human CM [14]–[16], and elevated levels of TNF in the cerebral spinal fluid correlated with encephalopathy in infected children [9]. Although inflammatory cytokines have been demonstrated to be critical in experimental CM, it is unclear if inflammatory cytokines have a causal role in human CM due to the difficulty of conducting mechanistic studies in humans. Parasite sequestration and inflammation can lead to endothelial activation and dysfunction. Widespread endothelial activation (including increased ICAM-1 expression and disruption of cell-junction proteins) has been observed in post-mortem studies of CM patients [1], [17], [18]. Biomarkers of endothelial activation and dysfunction, including soluble ICAM-1 (sICAM-1), von Willebrand factor (vWF) and its propeptide, and angiopoietin-2 (Ang-2) were elevated, while Ang-1 (a biomarker of endothelial quiescence) was decreased in severe and CM patients [19]–[25]. Further, impairment of endothelium-dependent vasodilation (a measure of endothelial dysfunction) correlated with disease severity in patients with malaria and was partially corrected by the administration of L-arginine [26].

Nitric oxide (NO) is a gaseous signaling molecule produced by a family of nitric oxide synthase (NOS) enzymes that catalyze the production of NO from L-arginine [27]. NO plays an important role in regulating endothelium function via multiple mechanisms, which include its action on the Ang-Tie2 system. The Ang-Tie2 system is a critical regulator of endothelial quiescence and activation, via the interaction of Ang-1 and Ang-2 with their cognate receptor Tie2. Ang-1 exerts anti-inflammatory effects and promotes endothelial quiescence, while Ang-2 promotes vascular permeability and endothelial activation [28]–[29]. NO can inhibit the release of Ang-2 from its storage site in endothelial cell Weibel-Palade (WP) bodies, and can induce the expression of Ang-1 [30]–[32]. NO also promotes endothelial quiescence by inhibiting inflammatory responses, decreasing the expression of cell adhesion molecules on the endothelium, and limiting intravascular platelet and leukocyte aggregation [33]–[34]. NO also acts as a vasodilator [35]. In the context of CM, these actions may reduce inflammation, decrease PE, platelet and leukocyte adhesion in the brain microvasculature, limit endothelial dysfunction, and improve cerebral blood flow [36].

Observations in children and adults with malaria suggest a correlation between impaired NO bioavailability and disease severity. Low NO levels, reduced NOS2 expression, low plasma concentrations of L-arginine, and increased levels of ADMA (an inhibitor of NOS) [26], [37]–[39], all have been associated with more severe disease.

*P. berghei* ANKA (PbA) infection in susceptible mice is a well described model that recapitulates several important features of human CM [40]–[43]. PbA-infected mice show signs of neurological dysfunction, including seizures, paralysis and coma. Systemic inflammation, brain hemorrhages, and vascular occlusion (by both leukocytes and PEs) have been observed in this model [40]–[43]. A recent report by White et al. [44] questioned the utility of the PbA-induced experimental CM model mainly based on an assertion that experimental CM is primarily driven by an immunological response involving little or no sequestration of parasites in the brain while human CM results from obstruction of microvessels in the brain by adhesion of PEs. However, this assertion has been challenged by several groups who agree that both processes are likely to contribute to CM pathogenesis in humans, and that the experimental CM model is a valuable tool for dissecting the mechanisms underlying the pathogenesis of CM that are not feasible to study in humans [41]–[43], [45]–[46].

Our objective for this study was to investigate whether inhaled NO (iNO) administration could reduce endothelial activation and protect mice from experimental CM. We chose to deliver iNO, rather than an NO donor, since this mode of NO administration does not produce systemic hypotension, is safe and easy to administer, and is already FDA-approved for use in infants for the treatment of respiratory failure, hypoxia, and pulmonary hypertension [47]. These properties make iNO an attractive candidate for transition to human trials.

We demonstrate that prophylactic administration of iNO to mice infected with PbA was associated with reduced systemic inflammation, endothelial quiescence, decreased ICAM-1 expression, reduced parasite accumulation in the brain, and preserved vascular integrity. Inhaled NO significantly improved survival when initiated in combination with artesunate as late as 5.5 days post infection.

## Results

### Inhaled NO prolongs survival in a mouse model of CM

To investigate the effects of iNO in experimental CM, mice were infected with PbA and administered gaseous NO continuously (80 ppm in air) in a prophylactic manner. NO_2_ levels were maintained at about 1.0 ppm, with peak NO_2_ levels never exceeding 2.0 ppm.

**Figure 1:**
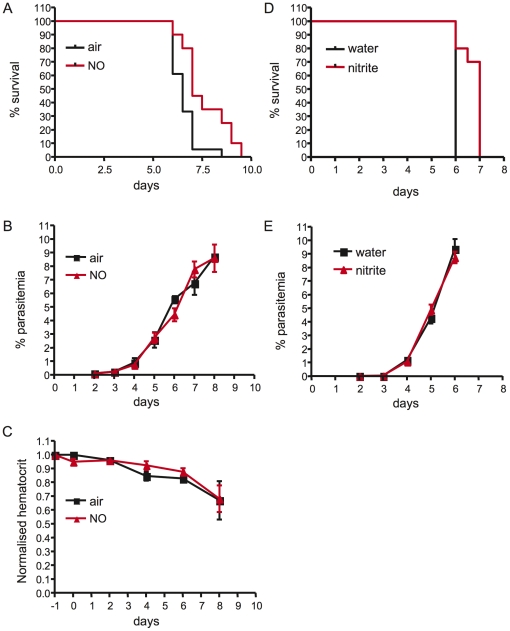
Prophylactic inhaled NO prolongs survival in mice infected with *P. berghei* ANKA. Kaplan Myer survival curves are shown for C57BL/6 mice infected with 1x10^6^ *P. berghei* ANKA PEs and treated in (A-C) with either 80 ppm NO (red) or air (black) starting one day prior to infection, or in (D-E) with either nitrite (administered in the drinking water at 500 mg/L of water, shown in red) or water (black). Survival (A and D) was assessed twice daily. Significant differences in survival were assessed by Log rank test. For (A) p = 0.0007, n = 18 in air and n = 20 in NO group, representative of 5 independent experiments. For (D) p = 0.0004, n = 10 per group, representative of 2 independent experiments. Parasitemia levels did not differ between the groups (B and E). Hematocrit did not differ between iNO- and air-treated groups (C).

**Figure 2:**
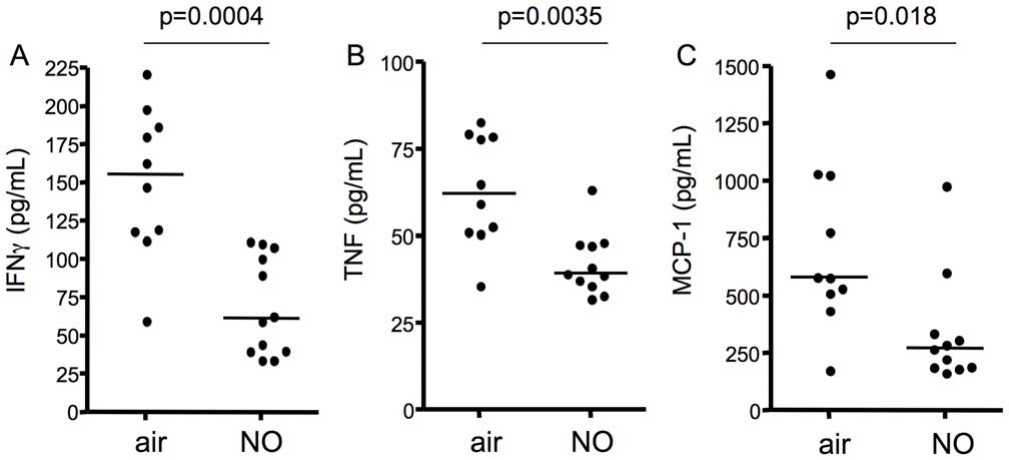
Inhaled NO reduces systemic inflammation in mice infected with *P. berghei* ANKA. Heparinized saphenous vein blood was collected on day 5 post-infection from mice that were prophylactically treated with either air or 80ppm NO. Plasma levels of (A) IFNγ, (B) TNF, and (C) MCP-1 were determined using the mouse inflammation cytometric bead array. Statistical differences were assessed by Mann-Whitney test. For (A) p = 0.0004, for (B) p = 0.0035, for (C) p = 0.018. Data are representative of 2 independent experiments. Plasma from uninfected mice was also tested as a control, and levels of all 3 cytokines were below the limit of detection.

Mice receiving iNO showed a modest but reproducible and highly significant enhancement in survival (p = 0.0007; Figure 1A) compared to control mice breathing air alone. Parasitemia (Figure 1B), weight (data not shown), and hematocrit (Figure 2C) did not differ significantly between the two groups.

Although NO itself is a short-lived molecule in the body with a half-life of less than one second, we observed systemic effects when NO was administered by inhalation in our model. Nitrite is a stable oxidation product of NO and was previously considered to be an inert metabolite of NO. However, recent evidence suggests that nitrite is a bioactive product that functions as an important vascular NO reservoir and endocrine transporter for NO [48]. Nitrite can generate NO by its reduction along a pH and oxygen gradient, serving as an acidic and hypoxic NO reservoir both in the vasculature and in tissue. To test the hypothesis that nitrite can function as an NO delivery system in our model, we administered nitrite to PbA-infected mice in their drinking water, starting on the day of infection. Mice receiving nitrite had a significant survival advantage over control mice similar to that observed with iNO (Figure 1D). As with iNO, parasitemia and weight did not differ significantly between control and mice receiving nitrite (Figure 1E and data not shown).

### Inhaled NO reduces systemic inflammation and endothelial cell activation

A dysregulated systemic cytokine response, characterized by high circulating levels of pro-inflammatory cytokines, has been associated with poor clinical outcomes in human malaria infection [49]–[51]. Mice susceptible to PbA also develop a marked systemic pro-inflammatory response during infection [52]. To determine if iNO could modulate malaria-induced inflammation, we assessed plasma levels of pro-inflammatory cytokines on day 5 post-infection. Compared to control mice, mice receiving iNO prophylactically had significantly reduced plasma levels of IFNγ, TNF and MCP-1 (p = 0.0004, p = 0.0035, p = 0.018 respectively; Figure 2). Elevated levels of these cytokines have previously been associated with poor outcome in human and murine models of malaria [49], [51], [52]. Thus, iNO was able to reduce malaria-associated systemic inflammation in experimental CM.

Vascular endothelium activation is also a central feature of both human and experimental CM [18]. The constitutive interaction between Ang-1 and Tie2 functions to maintain endothelium quiescence. Ang-2 antagonizes the Ang-1-Tie2 interaction, and sensitizes the endothelium to inflammatory stimuli such as TNF [53]. Elevated levels of Ang-2 and a higher Ang-2/Ang-1 ratio have been reported in patients with CM, as have other markers of endothelial activation including sICAM-1 and vWF [18]–[24], [54].

**Figure 3:**
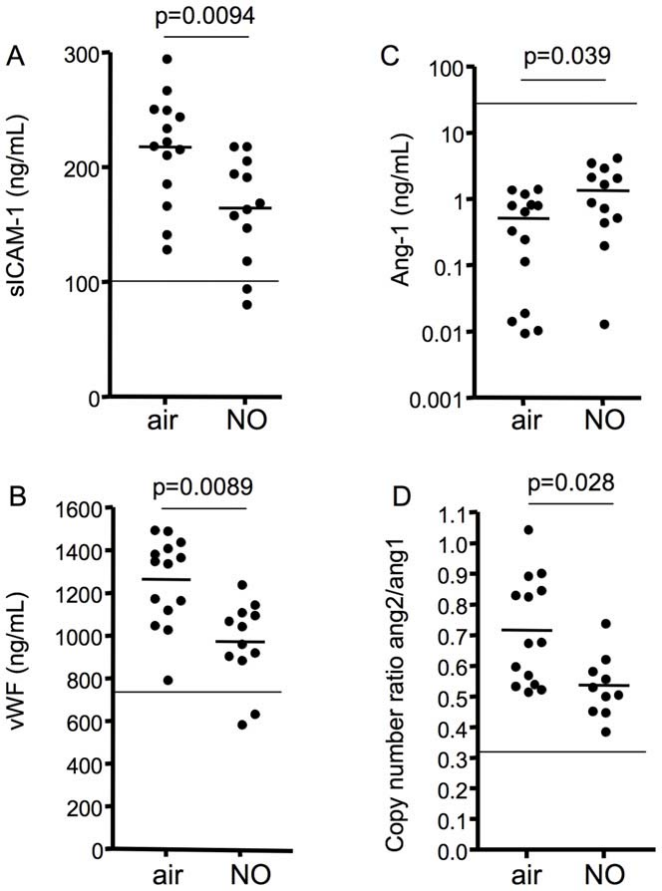
Inhaled NO modulates markers of endothelial activation in mice infected with *P. berghei* ANKA. (A-C) Heparinized saphenous vein blood was collected on day 5 post-infection from infected mice prophylactically treated with either air or 80ppm NO. Plasma levels of sICAM-1 (A), vWF (B), and Ang-1 (C) were determined by ELISA. The line represents median levels of these factors measured in uninfected mice. (D) Quantitative real time PCR was performed on RNA isolated from brains collected from parasitemia-matched air or 80ppm NO treated mice on day 6 post-infection. The copy number for ang-1 and ang-2 were determined and the ratio between ang-2 and ang-1 is shown. The line represents the copy number ratio in uninfected mice. Statistical differences were assessed using Mann-Whitney. For (A) p = 0.0094, for (B) p = 0.0089, for (C) p = 0.039, for (D) p = 0.028.

Levels of sICAM-1 and vWF were significantly reduced in mice receiving iNO (p = 0.0094 and p = 0.0089 respectively), suggesting less endothelial activation and a reduction in the exocytosis of WP bodies (Figure 3A-B). Levels of Ang-1 were significantly higher in iNO treated mice compared to those receiving air (p = 0.039; Figure 3C). Due to a lack of murine reagents, it was not possible to measure Ang-2 protein levels in plasma. To determine the effects of iNO on Ang-2, we measured Ang-2 and Ang-1 mRNA levels in brain homogenates of infected mice using quantitative real-time PCR (qRT-PCR). Mice treated with iNO had lower Ang-2/Ang-1 mRNA ratios than mice receiving air, indicating a shift in the Ang-2 to Ang-1 balance towards a more quiescent endothelium (p = 0.028; Figure 3D). Additionally, the ratio of plasma levels of vWF (also released from WP bodies) to Ang-1 were significantly lower in iNO treated mice (median ratio (IQR)  = 2.785 (1.067–83.56) for air vs. 0.7481 (0.135–2.17) for NO, p = 0.0289). Collectively, these data indicate that iNO reduced endothelial activation *in vivo* in this model.

### Inhaled NO reduces ICAM-1 expression and parasite accumulation in the brain

**Figure 4:**
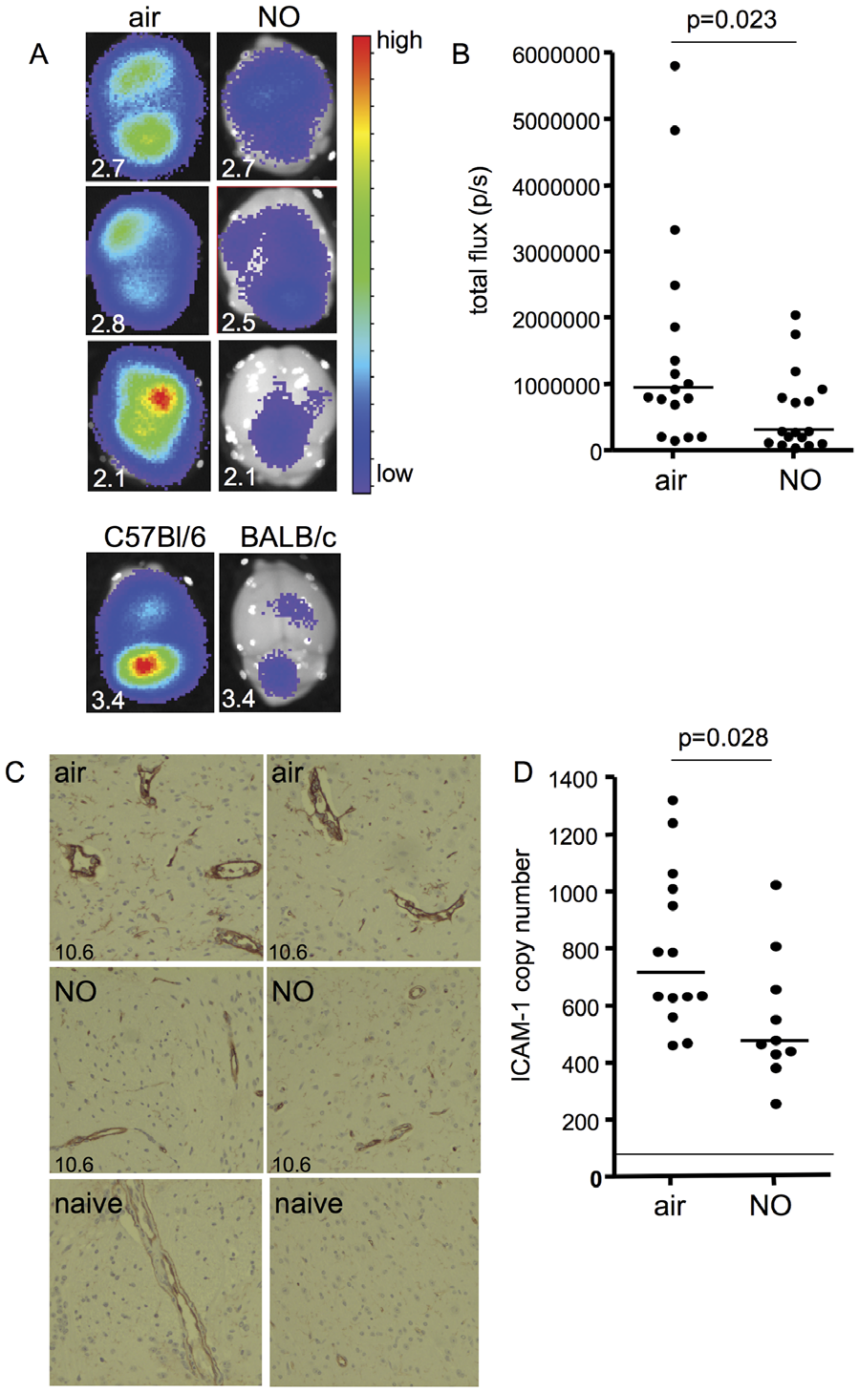
Inhaled NO reduces PE accumulation and ICAM-1 expression in the brains of *P. berghei* ANKA infected mice. (A) Luminometer imaging of brains collected on day 4 post-infection from mice infected with *P. berghei* ANKA–GFP–luciferase PEs and prophylactically treated with either air (left panels) or 80 ppm NO (right panels). The rainbow scale represents the relative level of luciferase activity (correlates with parasite load). The numbers on the lower left corner are the peripheral parasitemia levels for each mouse. The bottom panels are brains from a C57BL/6 mouse on the left and a BALB/c mouse on the right. (B) To quantify the brain bioluminescence, total flux (photons per second) was calculated for each brain. p = 0.023 by Mann-Whitney test, n = 17 per group. (C) Immunohistochemistry analysis of ICAM-1 on formalin fixed brain sections from parasitemia matched mice infected with *P. berghei* ANKA and prophylactically treated with either air (top panels) or 80 ppm NO (middle panels), or uninfected mice (bottom panels). (D) ICAM-1 mRNA expression displayed as copy number was quantified by quantitative real time PCR in the brains of parasitemia matched infected mice prophylactically treated with air or 80 ppm NO. The line represents the median copy number for uninfected mice. p =  0.028, by Mann-Whitney, n = 14 for air, n = 10 for NO.

Parasite sequestration in the cerebral microvasculature is frequently observed in post-mortem examinations of fatal cases of human CM, and is thought to be a contributing factor to the development of CM [1], [55]. The role of cerebral accumulation of PbA-PEs in experimental CM has been more controversial. Most blocked vessels during experimental CM contain a mixture of PEs and leukocytes, which suggests that PbA-PE accumulation alone may not be sufficient to cause blockage of microvessels in the brain during infection [56]–[58], [44]. However, accumulation of PbA-PEs has been observed in cerebral and cerebellar capillaries of mice displaying signs of experimental CM [56]-[57], [59]–[61]. To examine if iNO reduces PbA-PE accumulation in the brain, we performed luminometer imaging of the brains of mice infected with PbA expressing luciferase [62]. On day 4 post-infection, parasitemia-matched air- and iNO-treated mice were injected with luciferin (luciferase substrate), perfused to remove all blood and unbound PEs, and the brains were collected and imaged. Bioluminescence (assessed as total flux), an indication of PE accumulation in the brain, was significantly higher in control mice as compared to those receiving iNO, suggesting that although peripheral parasitemia was equal, iNO-treated mice had less PEs accumulating in the brain (p = 0.023; Figure 4 A-B). As a control, we imaged the brains of infected BALB/c mice. BALB/c mice are more resistant to experimental CM and do not develop neurological complications from infection [63], so one would expect to see less PE accumulation in the brain. In comparison with parasitemia-matched C57BL/6 mice, BALB/c mice had negligible bioluminescence detected in the brain suggesting a lack of PE accumulation (Figure 4A bottom panels).

To examine the potential mechanisms leading to reduced brain accumulation of PbA-PEs, we assessed ICAM-1 expression in the brains of iNO- and air-treated C57BL/6 mice. ICAM-1 is a cytokine-inducible cell surface receptor, and a major sequestration receptor for *P. falciparum* PEs in the brain [18], [64]. In clinical malaria, increased serum levels of the soluble form of ICAM-1 and increased protein expression in the microvascular endothelial sites have been observed [18]–[19], [64]–[67]. In mice, ICAM-1 has been shown to be upregulated by PbA infection [68]–[69], and ICAM-1 deficiency resulted in an improved survival from experimental CM, which was associated with abolition of blood-brain barrier breakdown, and a reduction in the accumulation of macrophages in the brain and PbA-PEs in the lung microvessels [70]. Using immunohistochemistry, we observed that ICAM-1 expression was upregulated in the brains of infected mice as compared to uninfected controls, with expression localized to blood vessels. Mice treated with iNO had lower levels of ICAM-1 expression in their cerebral vessels (Figure 4C). This finding is in agreement with previous observations on the effect of an NO donor on ICAM-1 protein levels in the brain of PbA infected mice [71]. To quantify the effect of iNO on ICAM-1 expression, we performed qRT-PCR on brain extracts. Consistent with the immunohistochemistry data, iNO treated mice had significantly lower levels of ICAM-1 mRNA in the brain as compared to air breathing controls (Figure 4D).

### Inhaled NO protects blood-brain barrier integrity

**Figure 5:**
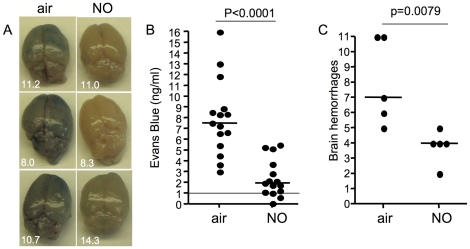
Inhaled NO prevents loss of blood-brain barrier vascular integrity in mice infected with *P. berghei* ANKA. (A) Photographs of brains from mice injected with Evans blue on day 6 post-infection. Brains from mice prophylactically treated with air (left panels) and with 80 ppm NO (right panels). The number on the lower left corner is the peripheral parasitemia for that mouse. (B) Quantification of total Evans blue extravasation in the brain as an indication of vascular leak. The line indicates median Evans blue values from uninfected mice. p<0.0001 by Mann-Whitney. n = 15 per group. (C) Number of hemorrhages quantified (blinded to the treatment group) from H&E stained sections of brains collected on day 6 post-infection from mice prophylactically treated with either air or 80 ppm NO. p = 0.0079 by Mann Whitney. n = 5 per group.

Because our data suggested that PE accumulation in the brain and endothelial activation were reduced in mice treated prophylactically with iNO, we investigated whether the integrity of the blood-brain barrier was also enhanced in PbA-infected C57BL/6 mice breathing NO. To assess vascular integrity, mice were injected with Evans blue on day 6 post-infection. Two hours post-injection, mice were euthanized, perfused to remove all blood, and the brains were assessed for Evans blue extravasation as a measure of vascular leak. iNO-treated mice had significantly reduced levels of vascular leak and preserved blood-brain barrier function as compared to air-breathing mice, despite having equivalent peripheral parasitemia (p<0.0001; Figure 5A-B).

To further investigate vascular integrity and neurological injury, we quantified (blinded to the treatment group) the number of brain hemorrhages on day 6 post-infection. iNO-treated mice had significantly fewer brain hemorrhages compared to controls (p = 0.0079; Figure 5C).

### Inhaled NO co-administered with artesunate improves survival over artesunate treatment alone

**Figure 6:**
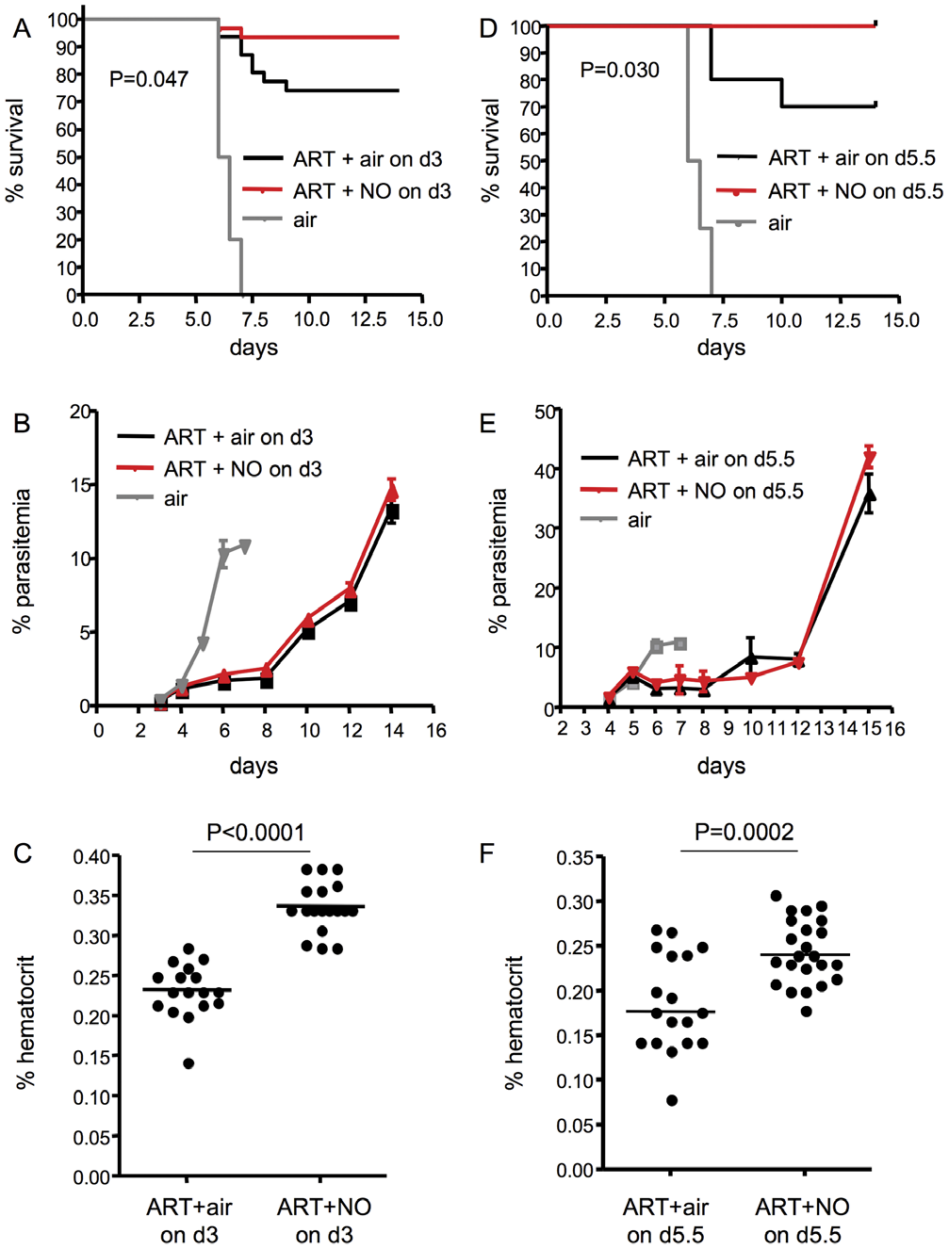
Inhaled NO administered in combination with artesunate confers a survival advantage over artesunate treatment alone in experimental CM. Mice were infected with *P. berghei* ANKA and were treated with either medical air (grey line), 10mg/kg artesunate (black line), or 10mg/kg artesunate plus 80 ppm NO (red line) starting on day 3 (A-C) or day 5.5 (D-F) post infection. Artesunate was given once daily by i.p. injection for 4 days starting on day 3, or once daily for one day starting on day 5.5. Air or 80 ppm NO was initiated on day 3 or day 5.5 and continued until the end of the experiment on day 15 post-infection. (A and D) Survival was monitored twice daily. Significant differences in survival were assessed by Log rank test. For Day 3: p = 0.049 for ART + air vs. ART + NO, n = 31 for ART + air, n = 30 for ART + NO, and n = 10 for air alone. For Day 5.5: p = 0.031 for ART + air vs. ART + NO, n = 10 for ART + air, n = 14 for ART + NO, and n = 8 for air alone. (B and E) Parasitemia did not differ significantly between the ART + air and the ART + NO groups. (C and F) Hematocrit was assessed on day 14 of infection in mice receiving ART + air or NO starting on day 3 (C) or 5.5 (F) post-infection. For Day 3: p<0.0001 for ART + air vs. ART + NO, n = 18 per group; for Day 5.5: p = 0.0002 for ART + air vs. ART + NO, n =  18 for ART + air, n = 23 for ART + NO; by Student's t-test. ART, artesunate, NO, nitric oxide.

Any adjunctive therapy for human CM would be administered in combination with anti-parasitic chemotherapy. Therefore, we next examined if iNO administered in combination with artesunate would confer improved survival over treatment with artesunate alone. PbA-infected C57BL/6 mice were given 4 once-daily doses of artesunate (10mg/kg/day) plus either air or 80 ppm iNO continuously starting on day 3 post-infection, or a single dose of artesunate plus either air or 80 ppm iNO continuously starting on day 5.5 post-infection. On day 3 of infection mice had detectable parasitemia but were not exhibiting any signs of CM. On day 5.5 of infection mice had experienced significant weight loss (56% of the mice had lost greater than 8% of their body weight) and were exhibiting the initial signs of CM, including hunched posture, ruffled fur, and lethargy (50% of untreated mice succumbed to their infection within 12 hours). Mice receiving iNO plus artesunate, starting on either day 3 (Figure 6A) or day 5.5 (Figure 6D) post-infection, had a significant survival advantage over mice receiving artesunate alone. Methemoglobin levels measured on day 7 post-infection did not differ between the two groups (normalized median (IQR); for ART + air  = 1.00 (0.76–1.19) and for ART + iNO  = 1.01 (0.95–1.17), p = 0.43 by Mann Whitney test, n = 11 per group). Of the mice that did not succumb to CM, hematocrit levels (Figure 6C and 6F) were significantly higher in mice receiving iNO compared to controls (although parasitemia did not differ between the two groups; Figure 6B and 6E).

## Discussion

Dysregulated host responses contribute to the adverse and fatal outcomes associated with severe malaria syndromes. Adjunctive therapies that target deleterious immune responses are a rational approach to improve outcome for life-threatening infections. However, relatively few adjunctive therapies have been tested for malaria, and even fewer have shown efficacy in clinical trials [22], [72]. Here, we report that prophylactic treatment with iNO reduced endothelial activation, inflammation, cerebral PE accumulation, and neurological injury, as assessed by vascular leak and CNS hemorrhages, in PbA-infected mice. In addition, therapeutic administration of iNO in combination with artesunate starting as late as day 5.5 post-infection conferred a survival advantage in an experimental model of CM. We propose that the attenuation of cerebral pathology in NO-breathing mice resulted, at least in part, from reduced inflammation and preservation of endothelial integrity.

In clinical malaria, impaired NO bioavailability and low plasma concentrations of L-arginine were associated with disease severity [26], [38], [73]. Also, recovery from severe malaria was accompanied by improvement in endothelial function and L-arginine level in the peripheral blood [22]. In experimental CM, restoring NO bioavailability by administering a NO-donor or L-arginine improves survival [71], [74]–[75]. Gragmalia et al. reported in data not shown that NO gas also had a protective effect [74]. Multiple factors contribute to the development of CM, including uncontrolled inflammation, accumulation of PEs in the brain, and blood–brain–barrier dysfunction. NO has been shown to regulate inflammatory responses and endothelial activation, to protect against vascular injury, and to improve cerebral hemodynamics, important protective processes in the context of CM [36], [71], [74], [75].

High levels of pro-inflammatory mediators correlate with disease severity and fatal outcomes in malaria infection. In agreement with the known anti-inflammatory properties of NO, malaria-infected mice receiving iNO had lower plasma levels of TNF, IFNγ and MCP-1, inflammatory markers associated with severe malaria [49]-[52]. Our data are consistent with a previous report that administration of an NO donor to PbA-infected mice reduced peripheral blood levels of the inflammatory biomarkers, IL-18 and soluble CD40 [74].

Inflammatory cytokines can contribute to endothelial activation by inducing the upregulation of adhesion molecules such as ICAM-1, a major receptor for *P. falciparum-*PEs. NO has a regulatory effect on endothelial cell adhesion molecule expression, helping to maintain a non-adhesive endothelium [76]. In *in vitro* studies, exogenous NO reduced the number of *P.falciparum* PEs adherent to endothelial cells by downregulating both basal and inflammation-induced expression of ICAM-1 [77]. We observed reduced accumulation of PbA-PEs in the brain of mice treated with iNO as compared to parasitemia-matched air-breathing mice, and this reduction was accompanied by decreased levels of ICAM-1 expression in the brain. This finding is in agreement with a previous study showing that administration of an NO donor reduced expression levels of ICAM-1 and PECAM and decreased leukocyte and platelet accumulation in the brain [71]. Our study confirms and extends these previous observations, suggesting that NO treatment may help to reduce cerebral sequestration of PEs, a key pathologic feature of CM. However, we have not tested whether PbA-PEs can directly adhere to the microvessels in the brain or whether ICAM-1 mediates the observed PbA-PE accumulation. The accumulation of PbA-PE may occur independently of ICAM-1. Recently, Bridges et al. proposed a novel mechanism whereby *P.falciparum* PEs can indirectly adhere to activated endothelial cells via platelets bound to large multimers of vWF [78]. Whether the same mechanism can be applied to the accumulation of PbA-PEs remains to be investigated. Endothelial activation is also associated with the exocytosis of WP bodies, which contain Ang-2, vWF, and its propeptide [30]. We, and others, have reported that levels of Ang-2 and vWF, and the ratio of serum/plasma Ang-2 to Ang-1 are quantitative and independent biomarkers of malaria severity and outcome [19]–[25], [54]. *In vitro*, NO inhibits the exocytosis of WP bodies, and thus regulates Ang-2 and vWF release [31]. We observed that mice receiving iNO had lower plasma levels of vWF, suggesting that the inhibitory effects of NO on WP body exocytosis may also occur *in vivo*. NO-treated mice also had higher levels of plasma Ang-1, and a lower Ang-2 to Ang-1 mRNA ratio in the brain. Ang-1 via its interaction with Tie2 can lead to vascular stabilization and decreased endothelial permeability [32], and may be a contributing factor (in combination with lower PE accumulation) to the reduction in brain hemorrhages observed in iNO-treated mice. Collectively, our data suggest that prophylactic administration of iNO reduced inflammation and endothelial activation, improved vascular stability, and decreased PE accumulation in the brain, leading to improved survival in experimental CM.

Prophylactic treatment with iNO alone conferred a significant but modest survival advantage in this model. Much of the potential therapeutic strategies tested in experimental CM including ablation or blockade of cytokine signaling were applied prior to the development of neurological signs, and were able to prevent, but not reverse the established CM pathologies, which is when they must be effective in clinical practice. In our study, NO improved outcome when administered in a more clinically relevant manner, as adjunctive therapy with artesunate even when initiated after the onset of neurological symptoms (5.5 day post-infection). iNO in combination with artesunate resulted in an increase in survival from 70% to 100% compared to treatment with artesunate alone. Mice receiving iNO also had higher hematocrit levels compared to mice receiving only artesunate. Oxidative damage to uninfected erythrocytes is thought to contribute to malarial anemia [79]–[80]. NO is a potent inducer of the hemoxygenase-1 and carbon monoxide pathway [81], which may serve to reduce oxidative damage and destruction of uninfected erythrocytes. Together, these data support the hypothesis that modulating the host immune response may represent an attractive adjunctive therapeutic strategy for CM, and encourages further testing of iNO in the clinical setting.

Clinically, delivering NO by inhalation offers several potential advantages. Inhaled NO is relatively easy to deliver (e.g. by face mask) in resource poor settings, does not require intravenous access, and can be quickly discontinued if adverse events are observed. In 1999 the US FDA approved the use of iNO (up to 80 ppm) for the treatment of neonates with hypoxic respiratory failure and based on broad clinical experience in hundreds of thousands of patients over the past 2 decades, has been shown to be safe and well tolerated even in critically ill patients [47], [82]–[84]. It is important to note that with administration of iNO it is recommended that levels of NO_2_ and methemoglobin should be monitored.

While its potential ease of delivery and safety make iNO an attractive candidate, its short half-life raise questions about its utility to exert systemic effects in more distant target sites such as the brain. Systemic effects of iNO have been observed in several *in vivo* models, and our study demonstrates that continuous inhalation of NO can effectively mediate systemic effects and attenuate some of the hallmark aspects of CM in the brain. iNO can exerts systemic effects via several possible mechanisms. iNO may bind to heme to form NO-heme, react with thiols to produces S-nitrosothiols, react with amine groups to form N-nitrosamines, all of which can regenerate NO in vascular beds [85]. iNO can also be converted into nitrite, which can convert back to NO in an acidic or hypoxic environment [48]. Administering nitrite improved survival in PbA-induced CM in this study, and via its reduction back to NO, has been shown to exert beneficial effects in cerebral ischemia-reperfusion injury, and LPS-induced shock [86]–[87].

In summary, our results using an experimental model of CM, suggest that iNO may be a promising adjunctive therapy for human CM. This hypothesis is currently under investigation in two independent randomized clinical trials in Uganda (one double-blind placebo controlled NCT01255215 [88]-[89], and one open label NCT01388842).

## Materials and Methods

### Murine model of experimental CM

*P. berghei* ANKA (PbA) was obtained from the Malaria Resource Centre (MR4, Bethesda MD) and maintained by passage in naïve mice.

The University Health Network Animal Use Committee approved all experiments. Male 7–9 week old C57BL/6 mice were purchased from Charles River. Infection was initiated by intraperitoneal injection of freshly isolated 1x10^6^ PbA PEs/mouse, and was monitored daily for up to 15 days by determining parasitemia on thin blood-smears stained with Diff Quik (American Scientific Products, Mississauga, ON). Weight and hematocrit were determined every other day. Methemoglobin levels were measured according to a previously published method [90], and were normalized to the air group median value. Mice were evaluated for signs of CM including limb paralysis, seizures, and coma, and were euthanized when moribund.

Treatment (air or iNO) was initiated either 1 day prior to or during infection. For experiments initiating treatment on day 3 post-infection, mice received 10mg/kg artesunate/day for 4 days and breathed air or NO beginning 3 days post-infection. For experiments initiating treatment on day 5.5 post-infection, mice received 10mg/kg artesunate/day for 1 day and breathed air or NO beginning 5.5 days post-infection. Mice were placed in flow-through chambers with free access to food and water, and received either medical air or 80ppm +/−5ppm NO mixed with medical air, continuously. Gas flow was at 10–12 L/min to minimize the concentration of NO_2_. Soda lime (200 g) was placed in each chamber to scavenge NO_2_. NO_2_ levels were maintained <1.5 ppm. NO and NO_2_ levels were measured using an AeroNOX device (Pulmonox Medical, Alberta, Canada).

For the nitrite experiments, nitrite (Sigma) was given in the drinking water (500mg/L made fresh daily) for the duration of the experiment, as described [86].

### Analysis of systemic inflammation and endothelial activation

Heparinized plasma was collected via the saphenous vein and frozen at −80°C. Plasma cytokine levels were assessed using the mouse inflammation cytokine bead array (BD bioscience, Mississauga, ON). Levels of Ang-1, sICAM-1, and vWF were determined by ELISA (R&D Systems, Minneapolis, MN).

### Analysis of vascular permeability

On day 6 post-infection, mice were injected intraperitoneally with 300 µl of 2% Evans blue. 2h post-injection mice were euthanized using isoflurane, and perfused with 20mls of PBS. Brains were collected, photographed, and placed in formamide for 48h to extract Evans blue. Evans blue was quantified using a spectophotometer at 605 nm and compared to a standard curve.

### Whole body imaging of parasite distribution

Mice were infected intraperitoneally with 1x10^6^ PEs of a luciferase-expressing PbA (PbGFP-LUC(con)) [62]. On day 4 post-infection, mice were anesthetized with isoflurane, injected with 100 µl of 5mg/ml D-luciferin (Caliper, Hopkinton, MA) intradermally, and imaged using the Xenogen IVIS Spectrum imager (Xenogen, Alameda, CA). Whole body images were taken 4 minutes post-injection, when the bioluminescence signal had reached its plateau. Mice were euthanized, and perfused (20mls PBS) to remove all the blood. Brains were collected and imaged 45 minutes post-luciferin injection. Bioluminesence was quantified using the Living Image 4.0 software. Total flux was used as a metric for comparison.

### Histological analysis

Brains were collected on day 6 post-infection, and fixed in 4% formalin for 3d. 4 µm slices were stained with H&E, or with a polyclonal antibody against ICAM-1 (R&D Systems, Minneapolis, MN), and examined blindly.

### Brain qRT PCR

RNA was extracted from snap frozen brain tissue after homogenization in TRIzol (1mL/100 mg tissue; Invitrogen, Burlington, ON) according to manufacturer's protocol. Extracted RNA (1 µg/sample) was treated with DNase I (Fermentas, Burlington, ON), and reverse transcribed to cDNA (BioRad, Mississauga, ON). cDNA was amplified in triplicate with SYBR Green master mix (Roche, Laval, QC) in the presence of 1 µM of forward and reverse primers in a Light Cycler 480 (Roche, Laval, QC). Transcript number was calculated based on Ct as compared to a standard curve of mouse genomic DNA included on each plate by Light Cycler 480 software (Roche, Laval, QC), and normalized by geometric averaging of GAPDH, HPRT and YWAHZ expression levels, as previously described [91]. The primer sequences (5′-3′) used are as follows: GAPDH, *F*-tcaacagcaactcccactcttcca, *R*-ttgtcattgagagcaatgccagcc; HPRT, *F*-ggagtcctgttgatgttgccagta, *R*-gggacgcagcaactgacatttcta; YWHAZ, *F*-agcaggcagagcgatatgatgaca, *R*-tccctgctcagtgacagacttcat; ANGPT1, *F*-cctctggtgaatattggcttggga, *R*-agcatgtactgcctctgactggtt; ANGPT2, *F*-agagtactggctgggcaatgagtt, *R*-ttcccagtccttcagctggatctt; ICAM, *F*-tggctgaaagatgagctcgagagt, *R*-gctcagctcaaacagcttccagtt.

### Statistical analysis

Survival studies were performed 5 times for air vs. iNO. Other experiments were repeated at least once. Statistical significance for survival studies was assessed by log-rank test. Other comparisons were assessed using Mann-Whitney test. A p-value of less than 0.05 was deemed significant. Statistical analyses were performed using GraphPad Prism software (LaJolla, CA).
